# Alimentary Canal.—On the Operation for Cleft Palate

**Published:** 1855-01

**Authors:** James Syme

**Affiliations:** Professor of Clynical Surgery in the University of Edinburgh.


### ARTICLE XV.

*Alimentary Canal.—On the Operation for Cleft Palate.* By JAMES SYME, Esq., F. R. S. E., Professor of Clynical Surgery in the University of Edinburgh.

[WE have inserted, in former volumes, several interesting articles on this subject, by Mr. Fergusson, (see *Retrospect*, vol. xiii, p. 141; vol. xxii, p. 409; vol. xxiii, p. 186; vol. xxv, p. 253.) We now give the opinion of another eminent surgeon; this will be seen to be opposed to that of Mr. Fergusson. Mr. Syme says that the alleged advantage of dividing the muscle of the palate, in the way done by Mr. Fergusson, is "an entire delusion." We must let Mr. Syme speak for himself. He says:]

The knife which Mr. Fergusson has recommended for the purpose should alone be sufficient to excite serious doubts as to the practicability of accomplishing the object in question; and if any one believes that he could divide completely the lax and yielding bellies of the palatal muscles by such means, his ideas of operative procedure must be very different from those which most anatomists would be led to entertain from their acquaintance with the animal tissues and the power of instruments upon them. That the muscles in question may be wounded or partially divided, I readily admit; but that they can be completely cut across, and still more, that they can be so with certainty, I no less positively deny. And if the division is only partial, it must be equally incompetent to produce the effect desired as an imperfect section of the adductor muscle of the eye-ball, is found to be for the cure of squinting.

The next point for consideration is the amount of advantage to be expected from division of the palatal muscles, supposing it were practicable. If they remain undivided, it is said there will be a constant tendency to separate the raw edges, and prevent union; while if they are divided, the palate being rendered lax and flaccid, will be in the most favorable condition. But it may here be asked, does the presence of muscular contractility in other situations impede union? In harelip, when the patient cries, the edges of the fissure are drawn so completly aside as nearly to efface the appearance of a lip; yet, if the operation be properly performed, no inconvenience is experienced from this source, and a sound union is accomplished with almost absolute certainty. In wounds of the eye-lids or cheek, even when attended with a considerable loss of substance, and when the raw edges are widely separated by the muscular contraction, no difficulty is found in keeping or uniting them together. It seems, indeed, as if, through some intuitive influence of the vis medicatrix, the muscles of the part concerned cease to contract with violence, and merely give that degree of tension or firmness which is well known to favor the adhesive process. On the other hand, there can be no doubt that if the palate admitted of being renderred perfectly lax and flaccid, it would then become very unfavorably situated, either for the performance of the operation, or for the accomplishment of a satisfactory result, since, as every practical surgeon knows, there is nothing more opposed to sound union between the surfaces of a wound, than the soft and flabby state which results from redundance or relaxation of the textures. From what has been said, it will, I trust, appear that complete division of the palatal muscles is impracticable,—that it is not requisite for a successful issue; and that, so far from being so, it would really be adverse to the object in view.

I may here remark, that very serious doubts have been entertained as to the expediency of performing this operation at all, since it cannot be attempted with any prospect of success until the patient is old enough to abstain from voluntary resistance; and then the organs of voice have become so practiced in overcoming the difficulties of their imperfect condition, that closure of the fissure, so far from being always beneficial, sometimes appears injurious to the distinctness of the articulation. But as the operation, if undertaken, should be conducted as far as possible to assure a satisfactory result in regard to the accomplishment of its immediate object, I shall now offer some remarks upon the different steps which are required on the occasion; and as it may be supposed, that although the principle of Mr. Fergusson's operation has seemed to me so questionable, there is perhaps something in the mode of its performance deserving of attention, it will be proper to inquire particularly into the method which he recommends.

Mr. Fergusson advises that, in the first place, the muscles should be divided by cutting on each side deeply into the pterygoid fossa with his triangular knife, and then using scissors to complete the process. Now, granting that the object in view may be thus accomplished, which I entirely disbelieve, it is at all events evident that the patient's throat must be in a painful, irritable and bleeding state when the next step is undertaken. But this step is of the most essential importance, since it is nothing less than placing the edges of the fissure in a condition admitting of their sound union, by paring them so smoothly and accurately, that without any undue loss of substance, the two lower surfaces may allow of perfect adaptation. How a process so nice and delicate is to be executed when the parts are obscured by blood, and the patient's power of self-command has been impaired or expended in enduring the much more painful preliminary proceeding, I am not at a loss to understand any more than the possibility of paring the edges of the fissure, as Mr. Fergusson advises, by seizing the uvula and cutting towards the commissure, especially with such a knife as the one he recommends for the purpose, which is thick and narrow instead of being thin and broad, as it ought to be. How the stitches are to be got in does not very clearly appear from Mr. Fergusson's description; but his advice to take them out on the second or third day, seems in the highest degree objectionable, since the union, however perfect in the first instance, can then have little power of resisting pressure, either from food or the tongue, independently of the disturbing influence of the pharyngeal muscles. The threads should penetrate the whole thickness of the palate, and be tied with no more force than is sufficient to retain the edges in contact, so that in the event of union taking place, they may neither cause sloughing of the portion included nor cut their way out by ulcerative absorption. In the case of a young lady, on whom I lately operated with such success that the adhesion was complete to the very tip of the uvula, one stitch was removed on the eighth and the other on the tenth day.

In performing the operation, the best way of proceeding is to place the patient on a chair in a good light, then to seize one end of the fissure at its middle by sharply pointed forceps, and introduce the knife, which should be thin and lancet-shaped, like the one of this form used for the extraction of cataract, a little above the commencement of the cleft, and cut evenly down from this point to the extremity of the uvula, so as to detach a slice of sufficient thickness to expose the submucous textures. The same process being repeated on the other side of the fissure, nothing remains but to introduce the stitches, which is best done by means of a slightly curved needle with fixed handle, which should be directed from without inwards, first on one side of the fissure, and then on the other. The two inner ends of the threads being then tied together, one of the other ends is to be pulled until the knot is drawn through the edge of the palate, and sufficiently far out of the mouth for the purpose in view. Two stitches are sufficient, one being placed at the root of the uvula, and the other midway between this point and the angle of the edges of the fissure. The threads should be tied with the "reef knot," and in doing so, resiliancy of the textures may be counteracted by keeping the threads in a state of tension. For at least two days the patient should subsist entirely upon fluids, and of these even have a very sparing allowance. He should also, of course, avoid talking, coughing, sneezing, and all other actions calculated to disturb the uniting process.—*Monthly Journal*, *April*, 1854, *p.* 298.—*Brathwaite's Retrospect.*

